# Thallium exists in opioid poisoned patients

**DOI:** 10.1186/s40199-015-0121-x

**Published:** 2015-08-01

**Authors:** Amir Ghaderi, Naser Vahdati-Mashhadian, Zohreh Oghabian, Valiallah Moradi, Reza Afshari, Omid Mehrpour

**Affiliations:** Department of Addiction studies, School of Medical Kashan University of Medical Sciences, Kashan, Iran; Departments of Pharmacodynamics and Toxicology, School of Pharmacy, Mashhad University of Medical Sciences, Mashhad, Iran; Department of Clinical Toxicology, Kerman University of Medical Sciences, Kerman, Iran; Addiction Research Centre, Mashhad University of Medical Sciences, Imam Reza Hospital, Ibn-e-Sina Street, Mashhad, 91735-348 Iran; British Columbia Center for Disease Control, Vancouver, Canada; Atherosclerosis and Coronary Artery Research Center, Birjand University of Medical Science, Birjand, Iran

**Keywords:** Thallium, Opioid, Poisoning

## Abstract

**Background:**

Thallium (Tl) is a toxic heavy metal that exists in nature. Tl poisoning (thallotoxicosis) may occur in opioid addicts. This study was designed to evaluate the frequency and level of urinary Tl in opioid abusers. In addition, clinical findings were evaluated.

**Methods:**

A total of 150 subjects were examined. Cases with a history of at least 3 years of abuse were admitted in the Imam Reza Hospital as the case group; 50 non-opioid abusers from the target population were included as the control group. Twenty-four hour urinary qualitative and quantitative Tl analyses were performed on both groups.

**Results:**

Out of the 150 subjects, 128 (85 %) were negative for qualitative urinary Tl, followed by 5 % (trace), 7 % (1+), 2 % (2+), and 1 % (3+). Mean (standard error (SE), Min–Max) quantitative urinary Tl level was 14 μg/L (3.5 μg/L, 0–346 μg/L). Mean urinary Tl level in the case group was 21 μg/L (5 μg/L, 0–346 μg/L) and that in the controls was 1 μg/L (0.14 μg/L, 0–26 μg/L), which were significantly different (*P* = 0.001). The most frequent clinical findings were ataxia (86 %), sweating (81 %), and constipation (54 %). In all cases (*n* = 150), the mean (SE) value for cases with positive qualitative urinary Tl was 26.8 μg/L (0.9 μg/L) and that in the negative cases was 2.3 μg/L (0.2 μg/L), which were significantly different (*P* = 0.002).

**Conclusions:**

This study showed that long-term opioid abuse may lead to Tl exposure. In opioid abusers with the clinical manifestation of thallotoxicosis, urinary Tl should be determined.

## Background

Thallium (Tl) is a highly toxic heavy metal with atomic number 81 and a molecular weight of 204.37 discovered by Sir William Crookes in 1861 [1]. It is a soft, bluish-white or gray water-insoluble metal but the salt forms are colorless, tasteless, and odorless. Tl is readily absorbed via ingestion, inhalation, and dermal contact [1–3]. Tl does not have any biological function in the body and the fatal dose is more than 10–15 mg/kg of body weight [4]. In the past, Tl was used as a hair remover and rodenticide because of its toxicity, but presently Tl administration is restricted to industrial usage such as optic lenses, and the radioisotope forms are used for medical purposes such as diagnosis of malignancies (such as melanoma) and tissue scintigraphy (such as cardiac scanning) [5]. Any amount of Tl in the body is abnormal [1]. In humans, Tl toxicity may occur occupationally (industrial exposure), accidentally (contact with Tl-containing material), or in cases of homicide; oral ingestion is the main route of Tl entrance in the body [1]. The clinical manifestation of thallotoxicosis has a wide spectrum but painful ascending peripheral neuropathy (both motor and sensory), gastrointestinal, and dermatologic manifestations (particularly alopecia) are major characteristics in Tl toxicity [5]. The exact mechanism of toxicity is still unclear, but because Tl ions are similar to potassium (K) ions, Tl may alter the number of K-dependent systems and enzymes in the body such as sulfhydryl groups, essential enzymes in the Krebs cycle, succinate dehydrogenase, and interactions with sodium-potassium ATPase and riboflavin [3, 6, 7]. Treatment includes early gastrointestinal decontamination, Prussian blue therapy (potassium ferric hexacyanoferrate), activated charcoal, laxatives, and enhanced elimination via forced diuresis and hemodialysis [3, 6, 8]. In Tl poisoning, early diagnosis and treatment is necessary for complete recovery; any delay may lead to permanent neurological disorders [6, 9]. Illicit opium may be adulterated with various substances such as sugars, quinine, caffeine, lactose, strychnine, paracetamol, and heavy metals such as lead [10, 11]. In fact, salesmen and smugglers may add any kind of heavy metals to opium to increase its weight for more profit. Recently, there have been reports of Tl toxicity in illicit opium users [12, 13]. We decided to evaluate Tl as an adulterant in opioid-like compounds from opium users, for the early diagnosis and treatment of thallotoxicosis.

## Methods

### Study design

This is a case–control study. One-hundred opioid-like abusers, raw opium, opium residue, crystal heroin, or mixed abusers for more than 3 years, aged 18–65 years, were recruited following admission to Imam Reza Hospital because of poisoning or rehabilitation as the case group. Controls were 50 non-abuser subjects with no history of drug use in the previous year. Urinary morphine tests using immunoassay tests were used to determine positive cases. Controls were verified to be opioid naïve via verbal conversation. All ethical approvals were considered and the aim of the study was explained to all subjects in both groups. Twenty-four hour urinary samples were taken on admission for qualitative and quantitative Tl analyses, and other data including demographic, neurological, dermal, and gastrointestinal clinical findings were entered into a predesigned checklist. Diphenoxylate, acetaminophen codeine, and tramadol users were excluded from the study.

### Laboratory assessment

In acute poisoning, high concentrations of Tl appear in the kidney; thus, urine is the fluid of choice for Tl detection [14]. The normal concentration is <5 μg/L (<24.5 nmol/L) in a 24-h urine sample. The urine samples were placed in 100 mL urine collection containers (polyethylene) and stored at −20 °C until analysis [15]. The first 24-h urinary samples in the two groups were examined for Tl using a qualitative (semi-quantitative) survey. Reagents included: 1) cyanide reagent: 1.6 g of sodium hydroxide, 1.2 g of sodium-potassium tartrate, and 1.36 g of potassium cyanide dissolved in 10 mL of water. 2) Dithizone solution (250 mg/L) in chloroform prepared fresh: 12 mL urine sample and 1 mL cyanide reagent to dithizone solution (dithizone + chloroform) were combined, shaken for 2 min, and left for 3–5 min. Two-phase formation occurred, consisting of an upper urine phase and a lower chloroform phase. A pink-red color in the lower chloroform phase indicates the presence of Tl and indicates a positive result. According to semi-quantitative Tl-screening tests, <50 μg/L is considered negative, trace (50–100 μg/L), 1+ (100–200 μg/L), 2+ (200–300 μg/L), 3+ (300–400 μg/L), and 4+ (>400 μg/L) [16]. In the second step, quantitative analysis via graphite furnace atomic absorption spectrometry (Perkin Elmer, Model 3030 with HGA 400 Programmer), which is a highly sensitive spectroscopic technique that provides excellent detection limits for measuring concentrations of Tl in urine, was performed on all samples [17]. In this test, we applied a Tl standard solution, traceable to standard reference materials from National Institute of Standards and Technology thallium(I) nitrate in nitric acid 0.5 mol/L (1000 mg/L) TlCertipur®, provided from Merck. To measure TL in urine, after extraction with nitric acid, sulfuric acid, and Triton X- 100, the liquid phases were separated from the sediment. Next, 2 % ammonium monovanadate in 1 % sodium hydroxide was then added to neutralize acidity to prevent graphite tube damage. The solution was centrifuged at high speed for 5 min, and 25 μL from the upper layer was injected into the graphite. A thermal program was selected for drying; 130 °C, 300 °C for organic solvents, and 800 °C for inorganic solvents and ash removal, and 1700 °C for atomization. Absorbance was measured at 276.8 nm [18, 19]. Detection limits were 0.2 μg/L, precision 3.65 %, and accuracy 97.4 % of analyses were determined by repeated analyses of biological reference material (SERONORM urine trace element level 2, lot 1011645) [20]. We evaluated both qualitative and quantitative urinary Tl because they complement each other.

### Statistical analysis

Data were analyzed with SPSS software (version 16). Student’s *t* test and Spearman’s coefficient test were used to analyze the data. *P* value was considered to be less than 0.05 throughout the study.

## Results

### Demographic findings

In total, 150 subjects, including 100 cases and 50 controls, were recruited. Among them, 134 (89 %) were male. Mean (standard error (SE), Min–Max) age was 41 (1.2, 19–85) years. Mean (SE) age in cases was 41 years (1.6) and in controls was 41 years (0.3), which were similar. Among cases, raw opium or opium residues were used by 66 % cases, followed by mixed (28 %), and crystal heroin (6 %). Cases only smoking were 49 %, those smoking and ingesting were 45 %, those only injecting were 3 %, and those using a mixture of methods were 3 %.

### Clinical findings

#### Neurological

Frequency of ataxia in cases (*n* = 100) was 86 %, followed by tremor (85 %), insomnia (83 %), depression (82 %), blurred vision (79 %), memory deficits (78 %), weakness (77 %), fatigue (77 %), aggressiveness (77 %), jerking movements (76 %), vertigo (73 %), emotional liability (65 %), headache (59 %), paresthesia (40 %), tinnitus (38 %), seizure (36 %), delirium-psychosis-coma (30 %), and choreoathetosis (0 %).

Dermal: Frequency of sweating in cases (*n* = 100) was 81 %, followed by scalp hair loss (45 %), dry skin (36 %), acne (8 %), rashes (6 %), body hair loss (5 %), Mees’ lines (0 %), and palmar erythema (0 %).

Gastrointestinal: Frequency of constipation in cases (*n* = 100) was 54 %, followed by nausea (20 %), vomiting (20 %), abdominal pain (16 %), and diarrhea (14 %).

### Qualitative urinary Tl results

For all subjects, 128 (85 %) were negative for qualitative urinary Tl, followed by 5 % (trace), 7 % (1+), 2 % (2+), and 1 % (3+). The distribution of qualitative results is shown in Table 1. The two groups were significantly different (*P* = 0.01).

**Table 1:** Qualitative urinary thallium results (*n* = 150)

Qualitative Urinary Thallium Results	Cases n (%)	Controls n (%)	Total n (%)
Negative	80 (80)	48 (96)	128 (85)
Trace	6 (6)	1 (2)	7 (5)
1+	10 (10)	1 (2)	11 (7)
2+	3 (3)	0 (0)	3 (2)
3+	1 (1)	0 (0)	1 (1)
Total	100 (100)	50 (100)	150 (100)

### Quantitative urinary Tl level

The mean (SE, Min–Max) quantitative urinary Tl level was 14 μg/L (3.5 μg/L, 0–346 μg/L). Mean urinary Tl levels in the case group were 21 μg/L (5 μg/L, 0–346 μg/L) and in controls were 1 μg/L (0.14 μg/L, 0–26 μg/L), which were significantly different (*P* = 0.001).

### Comparison of qualitative and quantitative results

In all cases (*n* = 150), the mean (SE) value for cases with positive qualitative urinary Tl levels was 26.8 μg/L (1 μg/L) and in negative cases was 2.3 μg/L (0.4 μg/L), which were significantly different (*P* = 0.002).

Duration of abuse was not associated with Tl levels (*r* = 0.329, *P* = 0.099). Positive Tl results were significantly higher for raw opium abusers in comparison with heroin abusers (*P* < 0.001). Table 2 summarizes these differences. Positive Tl results were significantly higher for smoke and ingestion compared with ingestion alone (*P* = 0.001). Table 2 summarizes these differences.

**Table 2:** Comparison of qualitative urinary thallium results in different type of abused drugs (*n* = 100)

type of abused drugs	Qualitative test						Total
	Negative	Trace	1+	2+	3+	Positive	
(Crystal) Heroin	5(83 %)	0(0 %)	1(16 %)	0(0 %)	0(0 %)	1(16 %)	6(100 %)
Opium	51(77 %)	5(7 %)	7(10 %)	2(3 %)	1(1 %)	15(21 %)	66(100 %)
Mixed user	24(85 %)	1(3 %)	2(7 %)	1(3 %)	0(0 %)	4(13 %)	28(100 %)
Total	80(80 %)	6(6 %)	10(10 %)	3(3 %)	1(1 %)	20(20 %)	100(100 %)

## Discussion

Our results indicate that contaminated opiates could be a source of Tl poisoning. Substance abuse is one of the most preventable health hazards worldwide. Iran appears to have both traditional and modern drug use and abuse problems, and it appears to play an important transit role in the transfer of opium and other drugs from Afghanistan to western countries [21, 22]. Adding heavy metals such as lead adulterant to the drugs is already described [23, 24]. Opium is still a most frequently abused drug. Informal, and often illegal, laboratories refine opium into a sticky, brown paste, which is pressed into bricks and sun dried. This material can then be ingested or smoked. This process results in introduction of impurities such as lead into the product [25]. Salesmen and smugglers may add any number of heavy metals to opium to increase the weight for more profit. Although the amount of Tl in opium is usually small, when taken in large amounts, opium adulterated with Tl can produce clinical toxicity. However, very few reports describe unusual and exotic causes of chronic Tl poisoning as an adulterant to illicit drugs [12–14]. Afshari *et al*. reported three cases of suspected Tl poisoning after heroin abuse. Urine qualitative tests resulted in a range from negative to 4+ Tl, and three months after substitution of heroin, signs and symptoms of thallotoxicos returned; they concluded that contaminated heroin was responsible [13]. Questel *et al.* also reported two cases of Tl poisoning in heroin users [12].

In our study, the most frequent symptoms were ataxia, tremor, insomnia, neuropathy (both sensory and motor), sweating, scalp hair loss, dry skin, constipation, nausea, and vomiting, which were slightly different from previous studies that emphasized on polyneuropathy, paresis, abdominal pain, and alopecia [4, 6, 9, 21]. This dissimilarity may be because of the amount of Tl used or less severity of toxicity. There were significant differences in urinary Tl tests between case and control groups in addition to clinical findings. Surprisingly, nearly one-third of our patients had a history of seizure, possibly because of abuse of tramadol or other unknown abuse of illicit drugs. In Iran, opium addicts have a great affinity for tramadol because of the euphoria experience or because of replacement of opiates [26–27]. Our study showed the most frequent illicit substance was raw opium and opium residue (66 %), and positive Tl results were significantly higher in this group in comparison with other abusers. In addition, ingestion and smoking was the most frequent route of consumption in the positive Tl group. Comparison between qualitative and quantitative results indicated that for exact diagnosis, it is better to incorporate both qualitative and quantitative analyses. Tl poisoning has a wide spectrum of clinical manifestation and any amount of Tl in the human body is abnormal. Moreover, it acts as a silent disease and diagnosis is sometimes difficult without suspicion. Our study showed that opium is a source of Tl in Iran and maybe in other parts of the world, so physicians should be aware of Tl exposure in opioid abusers, and they should be notified of its clinical symptoms. Although our findings suggested chronic Tl poisoning as an adulterant to illicit drugs, it should be noted that the findings may also result from cultivating opium poppies in Tl-contaminated soils or water.

## Conclusion

As Tl is an accumulative poison [9], any illicit abuser subject with neuropathy, gastrointestinal complaints, and alopecia should be suspected of thallotoxicosis, and both urinary qualitative and quantitative Tl analyses would be helpful for exact diagnosis.

### Limitations

This study is subjected to certain limitations because of the incontinency of opioid concentration and dose in the street drug abusers.
